# A functionally defined high-density NRF2 interactome reveals new conditional regulators of ARE transactivation

**DOI:** 10.1016/j.redox.2020.101686

**Published:** 2020-08-20

**Authors:** Jonathan Poh, Amy H. Ponsford, James Boyd, Jonathan Woodsmith, Ulrich Stelzl, Erich Wanker, Nicholas Harper, David MacEwan, Christopher M. Sanderson

**Affiliations:** a Institute of Translational Medicine, University of Liverpool, UK; b Institute of Pharmaceutical Sciences, Department of Pharmaceutical Chemistry, University of Graz, Austria; c Max-Delbrück Center for Molecular Medicine (MDC), Berlin-Buch, Germany

**Keywords:** NRF2/NFE2L2, KEAP1, Protein interaction network (PIN), Fluorescence cross-correlation spectroscopy (FCCS), Dual luminescence-based co-immunoprecipitation (DULIP), Human disease network, Binary interactome

## Abstract

NRF2 (NFE2L2) is a cytoprotective transcription factor associated with >60 human diseases, adverse drug reactions and therapeutic resistance. To provide insight into the complex regulation of NRF2 responses, 1962 predicted NRF2-partner interactions were systematically tested to generate an experimentally defined high-density human NRF2 interactome. Verification and conditional stratification of 46 new NRF2 partners was achieved by co-immunoprecipitation and the novel integration of quantitative data from dual luminescence-based co-immunoprecipitation (DULIP) assays and live-cell fluorescence cross-correlation spectroscopy (FCCS). The functional impact of new partners was then assessed in genetically edited loss-of-function (NRF2^−/−^) and disease-related gain-of-function (NRF2^T80K^ and KEAP1^−/−^) cell-lines. Of the new partners investigated >77% (17/22) modified NRF2 responses, including partners that only exhibited effects under disease-related conditions. This experimentally defined binary NRF2 interactome provides a new vision of the complex molecular networks that govern the modulation and consequence of NRF2 activity in health and disease.

## Highlights

• 1,962 predicted NRF2 partners were tested and 46 new binary partners were identified.
• 77% of tested new NRF2 partners modulate ARE-mediated gene expression.
• Some novel partners only exhibit functional effects under disease-like conditions.
• Quantitative evidence that KEAP1 can modulate ARE-transactivation independently of NRF2.
• New NRF2 binding partners reveal 130 novel disease gene associations.

## Graphical abstract

**Figure:**
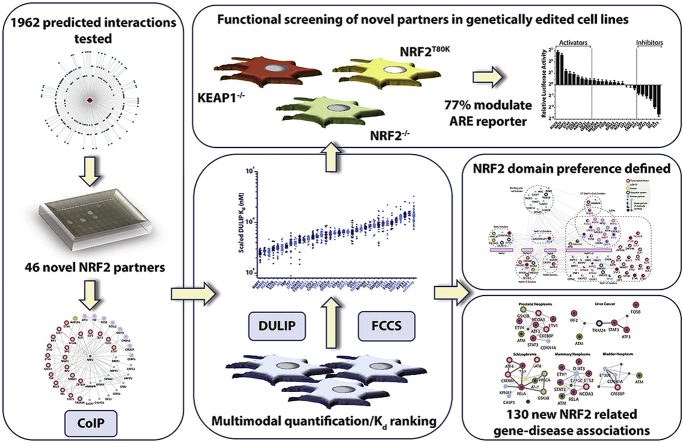

## 1 Introduction

The human *NFE2L2 (NRF2*) gene encodes a cap'n’collar (CNC) basic leucine zipper (bZIP) transcription factor. In addition to its well established role in redox homeostasis [1,2] there is increasing evidence that NRF2 has pleotropic effects on many cellular processes, driving expression of >240 functionally diverse genes [3]. The fundamental importance of the human NRF2 response network is emphasised by its association with >60 human diseases [4]. However, despite intense research, details of conditional regulation, functional-crosstalk or feedback within the human NRF2 network remain incomplete.

**Fig. 1:**
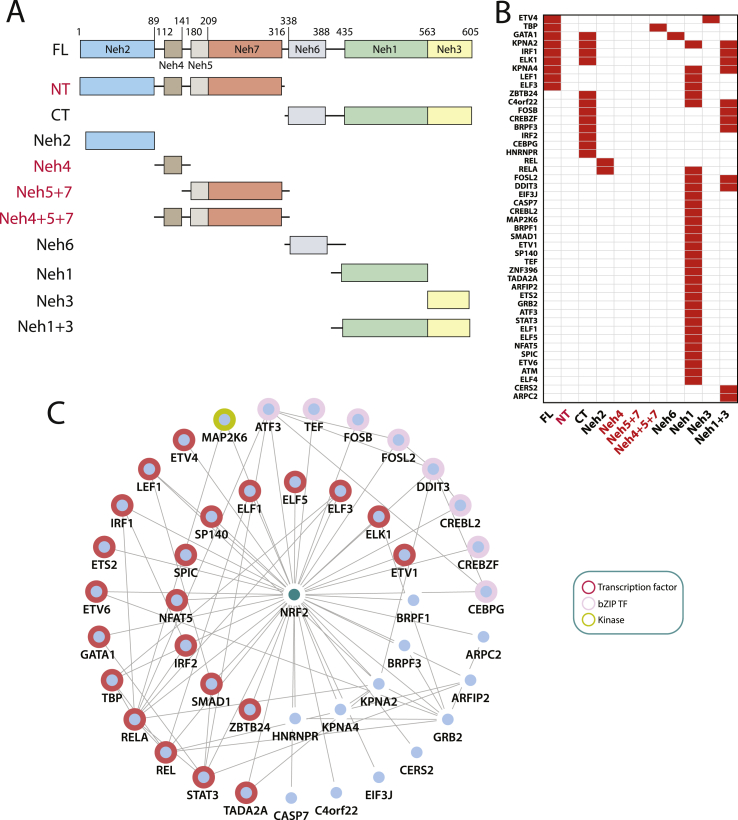
**Preliminary experimental screens of novel predicted NRF2 interaction partners**. (A) Human NFE2L2/NRF2-domain structure and fragments used in Y2H matrix screens. (B) Partner/NRF2-domain interaction preferences. (C) NRF2 binary interaction network showing all Y2H positive partners. Outer shell represents edges re-confirmed in co-immunoprecipitation assays. Transcription factors (TFs) are bordered in red, bZIP TFs in pink, and a kinase in light green. As the Neh4 and Neh5 domains tended to auto-activate Y2H reporters we were unable to define partner profiles for most of these regions. Auto-activating clones are shown in red. One Neh4+5+7 prey clone did not auto-activate and was used in the screen. (For interpretation of the references to colour in this figure legend, the reader is referred to the Web version of this article.)

At the protein level, NRF2 is composed of seven conserved NRF2-ECH (Neh) domains (Fig. 1A). Neh1 contains the CNC bZIP region, which facilitates heterodimerization with sMAF proteins (MAFG, MAFK, or MAFF) and binding to antioxidant response element (ARE) motifs in the promoter region of NRF2 target genes [5]. In contrast, the Neh2 domain mediates NRF2 degradation, primarily via the CRL3^KEAP1^ complex [6,7] but also via CRL4B^DCAF11^ [8]. The Neh6 domain also harbours degrons that facilitate SCF^β−TrCP^-mediated degradation [9,10]. Neh3, 4, and 5 facilitate interactions with transcriptional co-activators, such as chromodomain-helicase DNA binding protein 6 (CHD6) (Neh3 domain), CREB binding protein (CBP), and p300 (Neh4 and 5 region) [[11], [12], [13]]. Finally, the Neh7 domain mediates an interaction with the nuclear receptor retinoic X receptor alpha (RXRα), which represses ARE transactivation [14]. Given the functional differences between NRF2 domains, it is important to define where different partners interact, and their potential for competitive or conditional binding.

Under basal conditions NRF2 is sequestered in the cytoplasm through a direct interaction with KEAP1. This interaction leads to NRF2 ubiquitination and proteasomal degradation, ensuring low basal activity. Upon oxidative or xenobiotic insult, the KEAP1/NRF2 interaction is perturbed, preventing NRF2 degradation and allowing nascent NRF2 to drive transactivation of ARE-responsive genes, including *NQO1*, *GCLM*, and *HMOX1* [[15], [16], [17], [18], [19]]. However, there is increasing evidence that NRF2 activity can be modulated by other processes, including pro-inflammatory mitogen-activated kinase (MAPK) [20], nuclear factor kappa-B (NF-κB) [21], ER stress [5], and p53 response pathways [22]. In this context, it is conceivable that in order to maintain homeostasis, NRF2 may have evolved the ability to mediate the dynamic integration of responses from multiple stress response pathways. The diverse spectrum of NRF2 response genes and predicted interaction partners also implies a potential for complex mechanisms of conditional regulation and functional crosstalk with other biological processes.

Although induction of NRF2 counteracts the detrimental effects of inflammation [23], cardiovascular disease [24] and DNA damage [25], genetic mutations that constitutively enhance NRF2 activity are linked to therapeutic resistance and poor prognosis in several forms of cancer [[26], [27], [28], [29]]. As such, there is a need to develop new strategies to selectively modulate NRF2 responses under different pathological conditions. Unfortunately, lack of experimental evidence and systems-level analyses impedes mechanistic understanding of how NRF2 responses may be modulated, or the effects that changes in NRF2 activity may have on other cellular processes. Therefore, a more detailed functionally defined map of the human NRF2 interactome is required to inform development of improved therapeutic strategies. In addition to the 37 known binary human NRF2 interaction partners, a large-scale *in silico* study provided an intriguing insight into the potential complexity and diversity of the human NRF2 interactome, and its connectivity to other physiological and disease-related processes [30]. As a large proportion of these putative interactions were untested, we sought to generate a new high-density experimentally defined human NRF2 interactome, including verification, stratification, and functional analysis of novel partners. To maximise network coverage partner interaction screens were initially performed in high-stringency targeted yeast two-hybrid assays, which have the ability to detect weak yet functionally verifiable interactions [31,32]. In addition to conventional co-immunoprecipitation studies, a novel approach of combining live-cell fluorescence cross-correlation spectroscopy (FCCS) with high-sensitivity dual luminescence-based co-immunoprecipitation (DULIP) assays was used to calculate *in vivo* partner K_d_ values and stratify conditional partner preferences. Finally, a predictive analysis of biological function and disease association was performed to provide new insight into potential mechanistic links to human disease phenotypes.

## 2 Results and discussion

### 2.1 Experimental screening of predicted human NRF2 binary interaction partners

A speculative binary human NRF2 network was generated by prediction of domain-motif and domain-domain interactions using the Eukaryotic Linear Motif (ELM) and PRINCESS PPI-evaluation tools, as previously described [30]; with the inclusion of: (i) additional mammalian interologs from BioGRID [33,34], (ii) interologs involving the C. elegans NRF2 ortholog SKN-1 in Wormbase [35], WormNet [36], and the CCSB Worm ORFeome [37], (iii) interologs from the Drosophila melanogaster ortholog CncC in Flybase [38] and BioGRID databases, as well as (iv) predictions from the Reactome Functional Interactome (ReactomeFI) [39]. This resulted in a predicted network containing 187 potential binary NRF2 interaction partners (Supplementary Table S1); including 132 previously predicted NRF2 partners [30], together with additional non-mammalian interologs. In total, 133 of these putative binary NRF2 partners were cloned into yeast two-hybrid (Y2H) prey (pACTBE-B/pACTBD-B) and bait (pGBAE-B/pGBAD-B) expression vectors [40]. Following removal of auto-activating clones, 87 unique bait and 124 unique prey clones were generated (Supplementary Table S2). To identify NRF2 domain-specific interactions, fragments corresponding to individual and combined NRF2 Neh domains were generated, alongside full-length NRF2 (Fig. 1A). As constructs containing NRF2 Neh4 and Neh5 had a propensity to auto-activate it was not possible to use most of these clones in Y2H screens. However, one Y2H prey clone (Neh4+5+7) did not autoactivate and was included in the screen. Using all non-auto-activating clones, a total of 1962 binary interactions were systematically tested in a series of repeated Y2H matrix screens, resulting in the identification of 46 reproducible positive NRF2 partners, which show strong evidence of partner/domain specificity (Fig. 1B). Significantly, 28% (13/46) of the binary NRF2 partners identified in this screen were predicted interologs: seven of which were conserved in Drosophila, four in *C. elegans*, and two in mice, thereby justifying the inclusion of more distant orthologs in this study.

Initially, 43 positive NRF2 partners were expressed as mCherry-tagged constructs and tested by co-immunoprecipitation with EGFP-tagged NRF2. In total, 62.8% (27/43) of partners were reproducibly co-immunoprecipitated with EGFP-tagged NRF2 (Fig. 1C, Supplementary Fig. S1). As a high number of positive interaction partners were identified with the Neh1 or CT domains, it is possible that these proteins may act as conditionally competitive partners. Therefore, insight into the rank order of partner interaction strength, under basal and derepressed conditions, may aid prediction of conditional changes in partner preference. Significantly, a large proportion of new NRF2 partners were transcription factors (TFs), eight of which (ATF3, CEBPG, CREBL2, CREBZF, DDIT3, FOSB, FOSL2, TEF) belong to the same bZIP family as NRF2 (Fig. 1C); thus expanding the spectrum of NRF2 heterodimers, which have the potential to modulate ARE transactivation and target gene expression profiles.

**Fig. 2:**
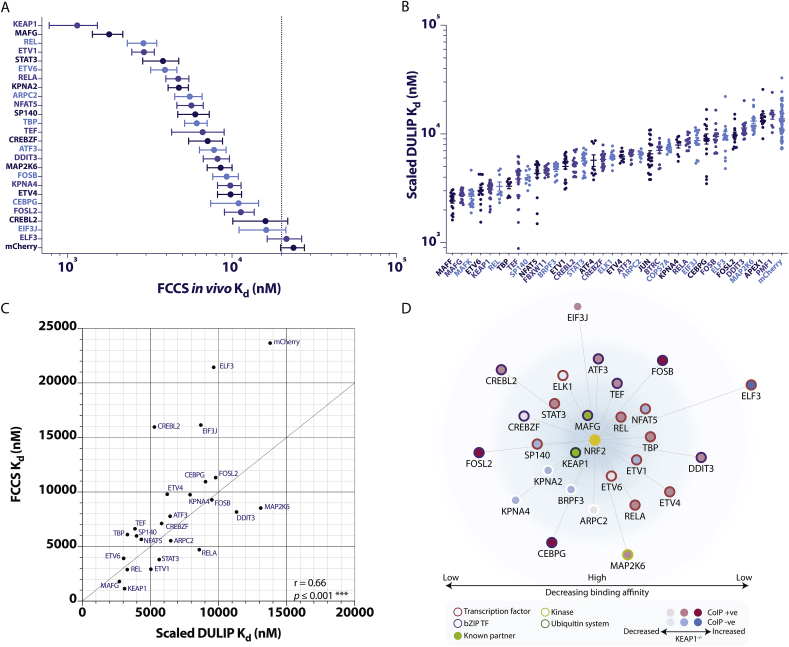
**Quantification of NRF2-partner binding strength**. (A) EGFP-NRF2 and 25 mCherry-tagged partners were co-expressed as pairs in HEK293T cells and interactions were measured in real time in live cells by fluorescence cross-correlation spectroscopy (FCCS). Binding strength is expressed as FCCS *in vivo* K_d_ values. Error bars represent 95% confidence intervals of the mean, n ≥ 30 single-cell measurements from ≥3 independent experiments for each combination. Threshold of positive binding is indicated by dotted line. (B) RL (*Renilla*-tagged)-NRF2 and 35 FL (firefly-tagged)-partners were co-expressed as pairs in HEK293T cells and interactions were quantified by DULIP. Binding strength is expressed as scaled DULIP K_d_ values ± SEM, n ≥ 9 measurements from ≥3 independent experiments. (C) FCCS K_d_ values are plotted against scaled DULIP K_d_ values. Correlation between FCCS K_d_ and scaled DULIP K_d_ data is represented by Pearson's r, *** indicates *p* ≤ 0.001. (D) Protein interaction network showing 27 NRF2 binary partners quantified by DULIP and/or FCCS. Partners confirmed by CoIP are shaded in red, negative partners are shaded blue. Shading intensity represents relative decrease or increase in binding affinity in KEAP1^−/−^ cells. Previously known partners are shaded in green. Transcription factors (TFs) are bordered in red, bZIP TFs in purple, a kinase in light green, and ubiquitin-related proteins in dark green. Edges represent binary NRF2-partner interactions. The network is visualised using Cytoscape in edge-weighted spring embedded layout. Length of edges are weighted by average normalised binding affinity from DULIP and FCCS experiments. (For interpretation of the references to colour in this figure legend, the reader is referred to the Web version of this article.)

Two complementary assays were used to compare the relative strength of NRF2-partner interactions. Binding affinities (*in vivo* K_d_ values) for KEAP1, MAFG and 23 novel NRF2 partners were first measured in HEK293T cells using fluorescence cross-correlation spectroscopy (FCCS) [41,42] (Fig. 2A). Values ranged over two orders of magnitude, with KEAP1 being the strongest binding partner, possessing an *in vivo* K_d_ of 1148 nM (95% CI, 776–1520 nM). This value is higher than the published *in vitro* K_d_ surface values of 167 and 580 nM for interactions between the NRF2 Neh2 domain or Neh2 peptide and KEAP1, respectively [43,44]. This difference may reflect the effects of co-complex or competitive binding events *in vivo* [42]. A large proportion of the partners, including KEAP1 and MAFG, have *in vivo* K_d_ values of 1000–10,000 nM, however five NRF2 partners (CEBPG, FOSL2, CREBL2, EIF3J, and ELF3) displayed very low binding affinities >10,000 nM (Fig. 2A, Supplementary Fig. S2-3). To re-assess these trends, the high-sensitivity dual luminescence-based co-immunoprecipitation (DULIP) method [45] was used to provide an independent measure of the relative strength and range of novel partner interactions. Six known binary NRF2 partners (MAFG, MAFK, MAFF, KEAP1, ATF4, and PMF1) and five known indirect or co-complex partners (BTRC, FBXW11, APEX1, COPS7A, JUN) were also included for comparison. As FCCS K_d_ and DULIP K_d_ values were highly concordant, all DULIP K_d_ values were scaled to equivalent molar concentrations via linear regression (Y = 0.213*X + 2.110, ***p ≤ 0.001) (Supplementary Fig. S4A, Supplementary Methods). Scaled DULIP *ex vivo* K_d_ values similarly spanned two orders of magnitude, with six novel partners (CEBPG, FOSB, ELF3, FOSL2, DDIT3, and MAP2K6) exhibiting weak binding to NRF2 (scaled DULIP K_d_ > 9000 nM). Significantly, known NRF2 partners exhibited a very similar range of binding affinities, ranging from very strong (KEAP1 and sMAF proteins) to very weak (PMF1 and APEX1) (Fig. 2B). The strong correlation between DULIP and FCCS K_d_ values are shown in Fig. 2C.

Given the dominance of KEAP1 binding under basal conditions, it is likely that other mechanisms of functional modulation will be most relevant when KEAP1/NRF2 interactions are genetically or conditionally disrupted. Therefore, the conditional hierarchy of NRF2-partners, and the potential for competitive dominance by KEAP1 was investigated by performing comparative DULIP assays in both WT and genetically edited KEAP1^−/−^ HEK293T cells. While the majority of interaction partners exhibit similar scaled DULIP K_d_ values in WT and KEAP1^−/−^ cells (Supplementary Fig. S4B), 13 partners exhibited a >1.25-fold difference between scaled DULIP K_d_ values in WT and KEAP1^−/−^ cells, with seven partners (ARPC2, ELK1, ETV6, CREBZF, ATF4, REL, and EIF3J) exhibiting a loss of affinity and six partners (JUN, CEBPG, PMF1, FOSL2, FOSB, and BTRC) showing a gain of affinity for NRF2 in KEAP1^−/−^ cells (Supplementary Fig. S4C). The resulting hierarchical binary NRF2 interaction network is shown in Fig. 2D. Considering these results, we propose that partners exhibiting lower K_d_ values in KEAP1^−/−^ cells may be competitively inhibited from binding NRF2 under basal conditions. However, these partners could in principle modulate NRF2 responses either: in the nuclear compartment where KEAP1 levels are low; in several forms of cancer, where NRF2/KEAP1 interactions are genetically perturbed; or under conditions of oxidative or xenobiotic stress, when KEAP1 mediated degradation of NRF2 is inhibited. It is also important to note that the rank order of NRF2 partner binding changes under derepressed (KEAP1^−/−^) conditions. This quantitative change in partner preference may provide a mechanism by which the extent or nature of NRF2 transcriptional responses can be conditionally tuned. In contrast, an increase in partner K_d_ values in KEAP1^−/−^ cells may suggest that under basal conditions, these partners could bind cooperatively with NRF2 and KEAP1 in multiprotein complexes. Quantitative data from this study also supports the current paradigm in which BTRC primarily governs nuclear degradation of NRF2 via CUL1-based proteasomal degradation under derepressed rather than basal conditions.

### 2.2 Phosphomimetic mutation of NRF2 Y576 inhibits ARE transactivation and alters bZIP binding preferences

**Fig. 3:**
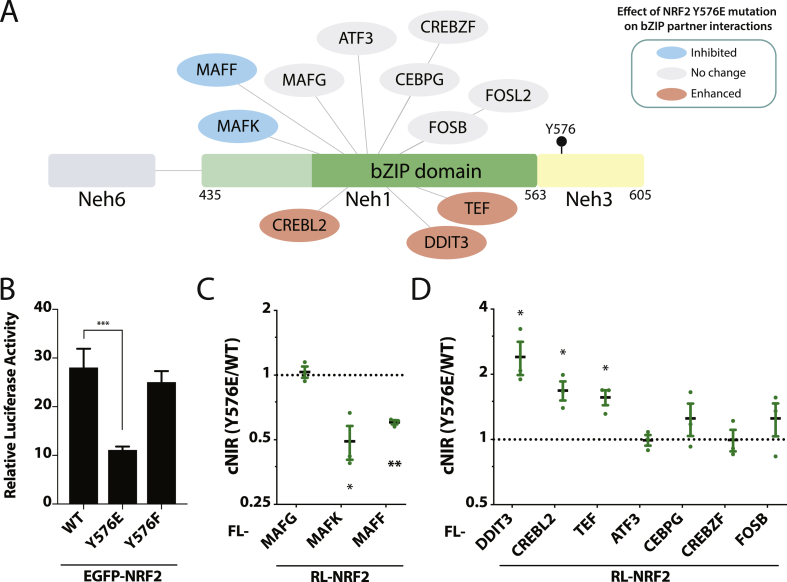
**Effect of NRF2 Y576 mutations on NRF2-bZIP partner interactions**. (A) C-terminal region of NRF2 showing the Y576 phosphorylation site in Neh3 and bZIP binding partners. Partner interactions that are inhibited or enhanced by the Y576E phospho-mimetic mutation are shown in blue or red, respectively. (B) Relative fold change of ARE-luciferase activity following 24 h transfection of NRF2 WT, Y576E, or Y576F, ***p ≤ 0.001; one-way ANOVA with Dunnett's multiple comparison post-hoc test; N = 3 independent experiments in triplicates. Change in binding strength of the NRF2 Y576E mutant compared to WT NRF2 for MAFG, MAFK and MAFF (C) or other NRF2 bZIP partners (D) in DULIP screens, *p ≤ 0.05, **p ≤ 0.005; one-sample *t*-test of log2-transformed corrected normalised interaction ratios (cNIR); N = 3 independent experiments in triplicates. RL, *Renilla*-tagged; FL, firefly-tagged. (For interpretation of the references to colour in this figure legend, the reader is referred to the Web version of this article.)

Although the Neh1 domain of NRF2 is known to contain the bZIP heterodimerization domain, quantitative DULIP assays show that both the Neh1 and Neh3 domains of NRF2 are essential for binding to MAFG (Supplementary Fig. S5A). This raises the possibility that changes within the Neh3 domain may have broader effects on the binding of other bZIP proteins in the neighbouring Neh1 region (Fig. 3A). A preliminary mutagenesis study of six predicted NRF2 phosphorylation sites S40, T80, S215, S408, T559 & Y576 [46] showed that only the phosphomimetic Y576E mutation significantly inhibited ARE transactivation (Supplementary Fig. S5B and Fig. 3B). As bZIP proteins are known to either facilitate or modulate NRF2 transcription, we performed a series of DULIP assays to investigate the effects of the Y576E mutation on bZIP protein recruitment. Interestingly, this mutation significantly inhibited interactions between NRF2/MAFK and NRF2/MAFF, while binding to MAFG was unaffected (Fig. 3C). As eight additional NRF2 bZIP-partners were identified in this study, the effects of the Y576E mutation on these interactions was also tested. While interactions with CREBZF, ATF3, CEBPG, and FOSB all remained unchanged, binding to TEF, CREBL2, and DDIT3 were significantly increased by the Y567E mutation (Fig. 3D).

These quantitative results provide the first evidence that the Neh3 domain of NRF2 is essential for heterodimerization with sMAF proteins. While the Neh3 domain may be required to maintain conformational integrity of the Neh1 region, our data indicates a possible new mechanism by which Y576 phosphorylation in Neh3 could change the rank order of bZIP partner binding, thereby providing a means by which NRF2 activity and target gene expression may be conditionally tuned.

### 2.3 Functional effects of new NRF2 partners on ARE transactivation

**Fig. 4:**
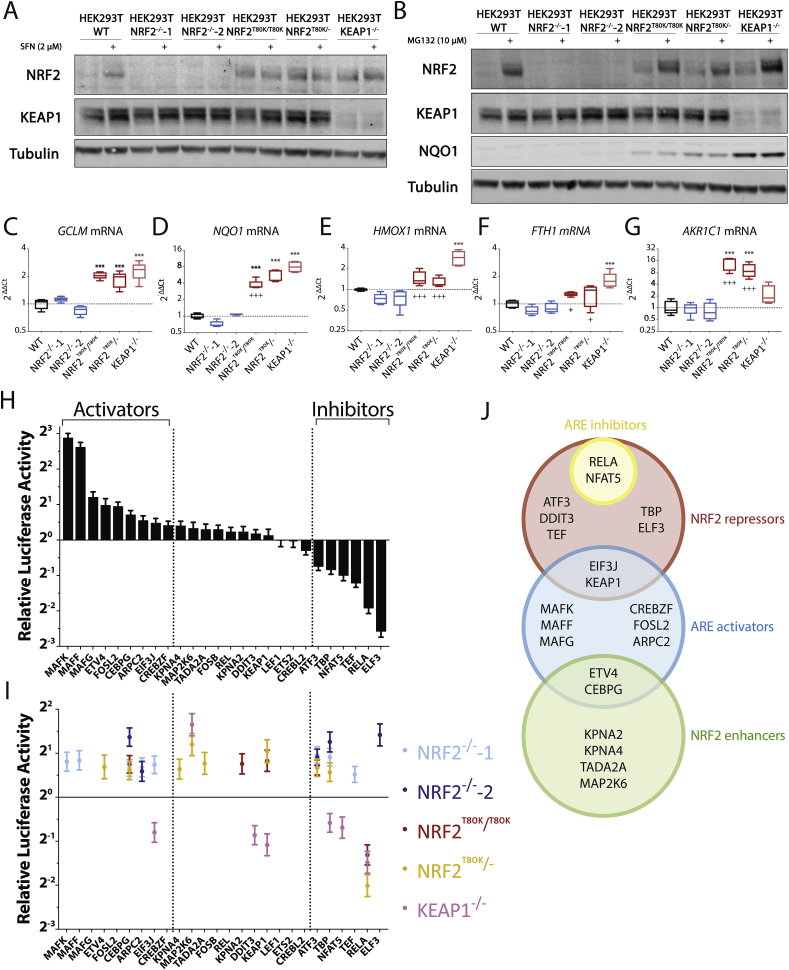
**Effects of new NRF2 partners on ARE transactivation activity**. Western blot images showing NRF2 protein levels in WT or genetically edited HEK293T cells in the presence or absence of sulforaphane (SFN) (A) or MG132 (B). Images are representative of three independent experiments. Transcript levels of *GCLM* (C), *NQO1* (D), *HMOX1* (E), *FTH1* (F) and *AKR1C1* (G) were evaluated by qPCR analysis to show relative levels of gene expression under untreated basal conditions. Statistical difference between ΔCt values was determined using one-way ANOVA with Dunnett's multiple comparison, ***p ≤ 0.001 against WT, ^+^p ≤ 0.05, ^+++^p ≤ 0.001 against KEAP1^−/−^; N = 5 independent experiments. Effects of interaction partners on log2-transformed normalised luciferase ratios are described by a linear mixed model fitted by residual maximum likelihood (REML). (H) Fold change in ARE-luciferase activity following 24 h transfection of interaction partner in WT cells. Values are expressed as estimates ± SE. (I) Fold change in ARE-luciferase perturbation in NRF2^−/−^-1, NRF2^−/−^-2, NRF2^T80K/T80K^, NRF2^T80K/^^−^, and KEAP1^−/−^ cells compared to WT cells. Only significant effects are displayed. Significance was evaluated using Welch-Satterthwaite t-tests; N ≥ 3 independent experiments in triplicates. (J) Classification of NRF2 interaction partners based on their effects on ARE transactivation.

Due to the dominant nature of the KEAP1/NRF2 interaction, the ability of new NRF2 partners to modulate ARE transactivation was analysed under both basal and derepressed conditions, using a series of CRISPR/Cas9-edited NRF2 gain-of-function (NRF2^T80K^ and KEAP1^−/−^) and loss-of-function (NRF2^−/−^) cell-lines. Evaluation of NRF2 levels following treatment with the NRF2 activator sulforaphane (SFN) or the proteasomal inhibitor MG132 confirmed that expression of NRF2 was abolished in NRF2^−/−^ cell-lines but enhanced in NRF2^T80K/T80K^, NRF2^T80K/-^ and KEAP1^−/−^ cells (Fig. 4A–B). Interestingly, comparative qPCR analysis of several NRF2 target genes under unstimulated conditions revealed differences in expression of *HMOX1*, *FTH1*, and *AKR1C1*, but not *GCLM* or *NQO1*, between KEAP1 binding-impaired (NRF2^T80K^) and KEAP1^−/−^ cell-lines (Fig. 4C–G). This shows that KEAP1 can influence ARE transactivation, and by extension NRF2 target gene expression, by some mechanism that is independent of its ability to bind NRF2. As KEAP1 is an E3 substrate adaptor these effects could result from changes in the degradation of other target proteins, which directly or indirectly affect the nature of NRF2 transcriptional responses. Having confirmed the functional phenotypes of each cell-line, ARE-luciferase reporter assays were performed to compare changes in ARE transactivation in response to ectopic expression of NRF2 interaction partners in HEK293T WT, NRF2^T80K/T80K^, NRF2^T80K/-^, KEAP1^−/−^ and two NRF2^−/−^ cell-lines. In total, the effects of 26 partners were examined in each of the six cell-lines. The resulting data was fitted using a linear mixed model; with the presumption that the effects of partners dependent on derepression of NRF2 would be significantly enhanced in the NRF2^T80K^ and KEAP1^−/−^ cell-lines, while the effects of partners specifically dependent on NRF2 would be dampened in the NRF2^−/−^ cell-lines.

Nine binary NRF2 partners (MAFK, MAFF, MAFG, ETV4, FOSL2, CEBPG, ARPC2, EIF3J, and CREBZF) significantly enhanced ARE-luciferase activity in HEK293T WT cells, while six partners (ATF3, TBP, NFAT5, TEF, RELA, and ELF3) caused inhibition compared to empty vector controls (Fig. 4H). ETV4 and CEBPG both exhibited a greater upregulation of NRF2 activity under derepressed conditions while TBP and NFAT5 caused greater downregulation in both NRF2^T80K^ and KEAP1^−/−^ cells. Significantly, RELA impaired ARE-luciferase transactivation in all three derepressed NRF2 cell-lines, demonstrating a striking conditional dominance under disease-like conditions. While ATF3 downregulated ARE-luciferase transactivation in both WT and KEAP1^−/−^ cells, this effect was absent in NRF2^T80K^ cells. This again emphasises the functional differences between KEAP1 ablation and selective genetic derepression of NRF2. Analysis of ARE perturbation in NRF2^−/−^ cell-lines shows that the effects of ATF3, TBP, TEF, and ELF3 are all NRF2-dependent (Fig. 4I). Interestingly, six partners (KPNA4, MAP2K6, TADA2A, KPNA2, DDIT3, and KEAP1) that did not perturb ARE-luciferase activity in WT cells, did induce significant changes in derepressed (NRF2^T80K^ and/or KEAP1^−/−^) cells. Of these, KPNA2, KPNA4, MAP2K6, and TADA2A all enhanced ARE-luciferase activity. Despite being one of the weakest NRF2 binding partners MAP2K6 displaying a strong dependence on disease-related NRF2 derepression; with effects being seen in all three derepressed NRF2 cell-lines. Significantly, ectopic expression of KEAP1 increased ARE-luciferase transactivation in both T80K cell-lines, confirming that KEAP1 can indirectly enhance ARE transactivation, via an NRF2-independent mechanism. These functional screens demonstrate that the new high-density binary NRF2 interactome contains novel activators and inhibitors of ARE transactivation, as well as a subset of partners that specifically exhibit NRF2-dependent effects under disease-related conditions (Fig. 4J). Finally, it is important to note that several of the partners that modulate ARE-luciferase transactivation correspond to very weak NRF2 binding partners.

### 2.4 Predictive analysis of the expanded human NRF2 interactome

**Fig. 5:**
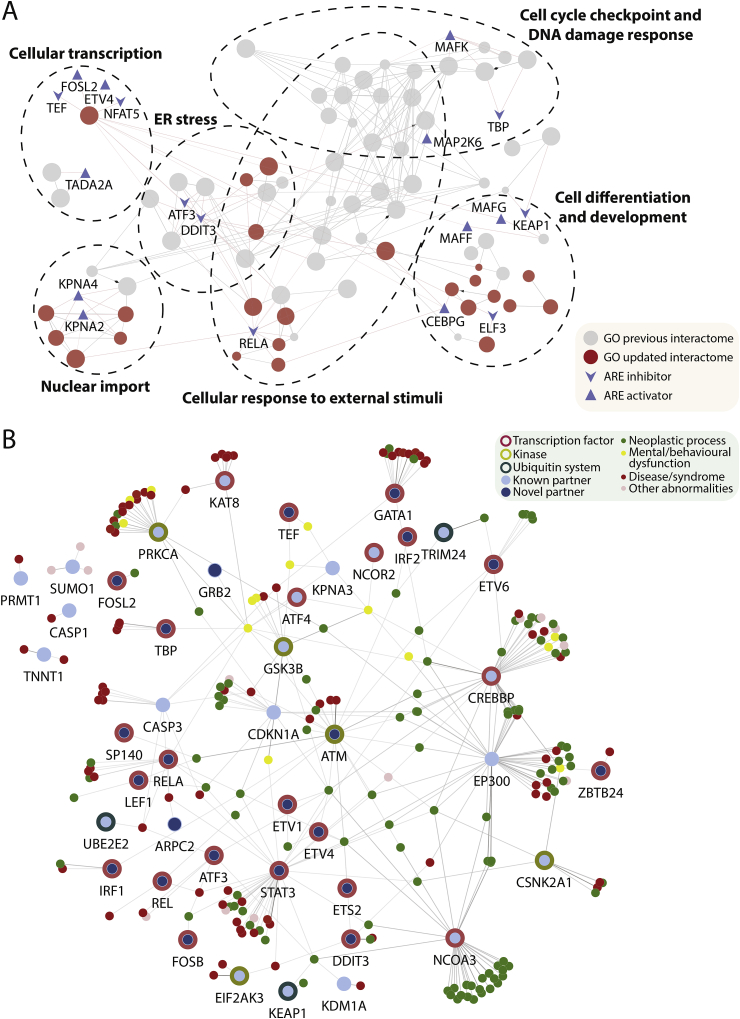
**Functional and disease association landscape of NRF2 binary partners**. (A) Network showing Gene Ontology Biological Process (GO BP) terms between levels 3 and 6 that are significantly enriched in NRF2 binary partners, using a cut-off of Bonferroni step-down *p-*value ≤ 0.05 in a two-sided hypergeometric test using the Cytoscape ClueGO plugin. Nodes represent GO BP terms grouped according to the degree of overlap in gene-set using a kappa score of 0.5. Node size is proportional to number of associated genes. Nodes are shaded grey if significantly enriched (>50%) in the previous interactome and red if selectively enriched in the new high density NRF2 interactome. Blue nodes represent NRF2 binary partners that either inhibit or activate ARE transactivation. Red edges represent protein-term interactions and grey edges represent term-term interactions. (B) Disease-gene association network for NRF2 binary partners using a cut-off ≥ 0.2 based on the DisGeNET tool. Transcription factors are bordered in red, bZIP TFs in pink, kinases in light green, and ubiquitin-related proteins in dark green. Previously known partners are shaded in light blue and novel partners from this study in dark blue. Diseases nodes are shaded green if representing a neoplastic process, yellow if representing a mental or behavioural process, red if representing a disease or a syndrome, and pink for other abnormalities. Edges represent gene-disease interactions. (For interpretation of the references to colour in this figure legend, the reader is referred to the Web version of this article.)

Functional annotation of the expanded binary interactome was performed using Gene Ontology Biological Processes (GO BP) with biological processes being grouped according to functional class, using the Cytoscape ClueGO plugin [47] (Fig. 5A). In total, 76 GO BP terms (grouped into 18 functional classes) were significantly enriched; including four previously identified themes (ER stress, cellular response to external stimuli, cellular transcription, and cell cycle/DNA damage response) and two additional themes (nuclear import, and cell differentiation/development) identified in this study. Considering novel partners that significantly alter ARE transactivation, ATF3 and DDIT3 (both NRF2 repressors) were over-represented in GO BP terms involved in ER stress, thus highlighting a novel intersection between oxidative and ER stress responses (Fig. 5A).

An analysis of disease association was also performed on the new expanded human NRF2 interactome. In total 334 gene-disease associations were identified, including 130 (39.9%) from 22 (51.2%) newly identified NRF2 partners (Fig. 5B, Supplementary Table S3). Six of the new NRF2 partners associated with known NRF2-disease phenotypes (Table 1, Supplementary Fig. S6A), and 13 disease phenotypes involved four or more binary NRF2 partners (Table 2). Given the potential for functional co-operativity between NRF2 partners, the DisGeNET network was merged with a PPI network constructed from five publicly available human PPI databases (BioGRID [33,34], IntAct [48], HPRD [49], HuRI [50], and InnateDB [51]). Disease-centric sub-networks involving two or more NRF2 partners were then extracted, excluding partner pairs with known physical association (Supplementary Fig. S6B). Interestingly, STAT3 and ATM were jointly associated with five different NRF2 disease phenotypes (pancreatic neoplasm, squamous cell carcinoma, stomach neoplasms, prostatic neoplasms, and mammary neoplasms), suggesting possible mechanistic similarities in the pathophysiology of these diseases.

**Table 1:** Disease phenotypes associated with NRF2.

Disease	UMLS CUI	NRF2 binary partners associated
Squamous Cell Carcinoma	C0007137	**ATM**, CREBBP, CSNK2A1, EP300, NCOA3, **STAT3**
Non-Small Cell Lung Carcinoma	C0007131	**ATF3**, KDM1A, KEAP1, NCOA3, **STAT3**
Squamous Cell Carcinoma of Oesophagus	C0279626	CDKN1A, CREBBP, EP300
Liver Neoplasms	C0023903	**ATF3, FOSB, STAT3**
Liver Carcinoma	C2239176	ATM, IRF2, TRIM24
Skin Neoplasms	C0037286	CSNK2A1
Diabetic Nephropathy	C0011881	**RELA**
Pulmonary Fibrosis	C0034069	–
Non-Alcoholic Fatty Liver Disease	C0400966	–
Hyperglycaemia	C0020456	–
Acute Kidney Injury	C2609414	–
Acute Lung Injury	C0242488	–
Kidney Diseases	C0022658	–
Liver Cirrhosis	C0023890	–
Gastrointestinal Diseases	C0017178	–

*Novel partners are highlighted in bold.

**Table 2:** Disease phenotypes associated with multiple NRF2 binary partners.

Disease	UMLS CUI	NRF2 binary partners associated
Schizophrenia	C0036341	ATF4, **ATM**, CASP3, CREBBP, **GRB2**, GSK3B, KAT8, KPNA3, PRKCA, **RELA, TBP**
Prostatic Neoplasms	C0033578	**ATF3, ATM**, CDKN1A, CREBBP, **ETV1, ETV4**, GSK3B, **STAT3**
Mammary Neoplasms	C1458155	**ATM, DDIT3**, EP300, **ETS2, ETV4, RELA, STAT3**
Acute Myelocytic Leukaemia	C0023467	CREBBP, EP300, **ETV6, GATA1, STAT3**
Squamous Cell Carcinoma	C0007137	**ATM**, CREBBP, CSNK2A1, EP300, **STAT3**
Stomach Neoplasms	C0038356	**ATM**, CDKN1A, **IRF1, STAT3**
Ulcerative Colitis	C0009324	**ARPC2**, CASP3, **RELA, STAT3**
Colonic Neoplasms	C0009375	CDKN1A, **LEF1, RELA, STAT3**
Bipolar Disorder	C0005586	ATF4, CREBBP, GSK3B, NCOR2
Diabetes Mellitus, Non-Insulin-Dependent	C0011860	**ATF3**, CASP3, **RELA**, UBE2E2
Non-Small Cell Lung Carcinoma	C0007131	**ATF3**, KDM1A, KEAP1, **STAT3**
Bladder Neoplasm	C0005695	**ATM**, CDKN1A, CREBBP, EP300
Intellectual Disability	C3714756	CREBBP, CSNK2A1, EP300, **ZBTB24**

*Novel partners are highlighted in bold.

## 3 Conclusions

**Fig. 6:**
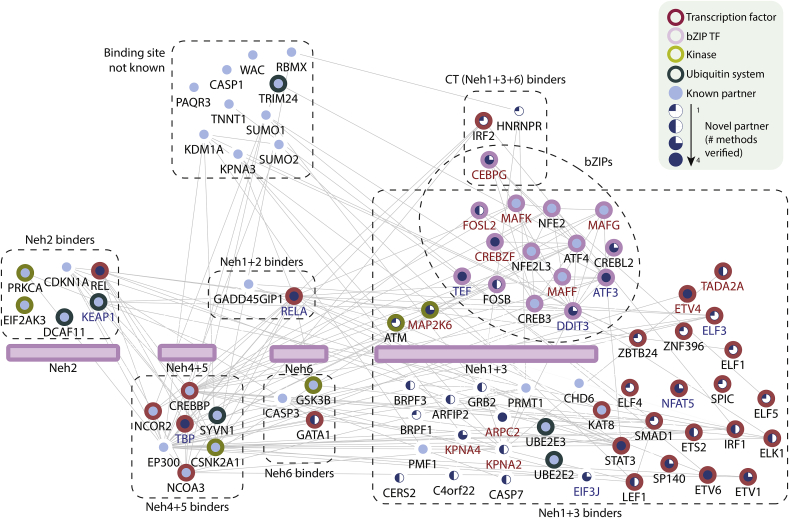
**New high-density binary NRF2 interactome**. Previously known partners are shaded in light blue and new partners from this study in dark blue. The area of shading within nodes corresponds to the number of techniques used for confirmation in this study. ARE activators are highlighted in red and ARE inhibitors in blue. Transcription factors are bordered in red, bZIP TFs in pink, kinases in light green, and ubiquitin-related proteins in dark green. Edges represent connectivity between proteins based on an in-house generated interactome. NRF2-partner edges are not displayed. Partners are grouped to show NRF2 binding domain. (For interpretation of the references to colour in this figure legend, the reader is referred to the Web version of this article.)

Although NRF2 is known to play a major role in redox homeostasis, the complex mechanisms by which NRF2 responses are regulated cannot be explained by current knowledge of well characterised pathway components. This study represents the first large-scale quantitative analysis of the human NRF2 protein interaction network. It was designed to provide a broader insight into the diverse range of cellular components that interact directly with the different functional domains of NRF2. Using a combination of high stringency protein interaction assays we identified 46 novel binary NRF2 partners (Fig. 6). Significantly, >77% of the novel partners tested were shown to effect NRF2 driven ARE-transactivation. Quantitative analysis of protein interactions enabled the rank ordering and strength of partner interactions to be defined, both *in vitro* and in live cells. These data confirm the dominance of KEAP1 and sMAF proteins under basal conditions, but reveal an emerging model of regulation in which changes that disrupt binding of dominant basal partners (KEAP1 and sMAF proteins) allow weaker binding partners to interact with NRF2, thereby facilitating changes in transcriptional activity. In addition, we provide the first evidence that the NRF2 Neh3 domain is essential for recruitment of MAFG, and demonstrate that a phosphomimetic Y576E mutation not only inhibits ARE transactivation, but also selectively inhibits interactions with MAFK and MAFF, while enhancing interactions with other bZIP partners. Finally, we present quantitative data to show that KEAP1 can influence ARE transactivation in an NRF2 independent manner.

These data significantly expand the range of candidate proteins that have the potential to modulate NRF2 responses. Together they support an emerging paradigm that NRF2 acts as a dynamic sensor to facilitate communication and balance between multiple conditional stress-responses. This new high-density binary NRF2 interactome provides a resource to inform future investigation into the complex conditional regulation of NRF2 responses, and the development of better rationally designed strategies for therapeutic intervention or drug sensitisation.

## 4 Material and methods

### 4.1 Lead contact and materials availability

Further information and requests for resources and reagents should be directed to and will be fulfilled by the Lead Contact, Christopher M Sanderson (, cmsand@liverpool.ac.uk).

### 4.2 Experimental model and subject details

#### 4.2.1 Cell-lines

Human embryonic kidney (HEK293T) cells were obtained from ATCC. All parent and derived HEK293T cell-lines were cultured in DMEM with GlutaMAX supplemented with 10% FBS, 1% NEAA, and 1% pencillin/streptomycin (Invitrogen) in a humidified atmosphere of 37 °C and 5% CO_2_.

### 4.3 Method details

#### 4.3.1 Generation of bait and prey yeast two-hybrid clones

Generic primers containing Gateway flanking sequences were designed to amplify any ORF from pDONR201/221/223 vectors by proof-reading PCR to facilitate *in vivo* gap repair cloning into Gateway compatible BamHI linearised bait (pGBAD/E-B) and prey (pACTBD/E-B) Y2H vectors [40]. This approach allowed all clones where possible to be expressed as both bait and prey fusions. Gap repair reactions were performed as previously described [52]. Y2H assays utilised the bait PJ69-4a yeast strain and prey PJ69-4α strain.

#### 4.3.2 Yeast two-hybrid matrix screens

NRF2 clones were systematically mated against the array of predicted interaction partners in both bait/prey orientations where possible. The Neh4 and Neh5 domains of NRF2 showed a propensity to auto-activate in Y2H assays, however this was not the case for the combined Neh4+5+7 domains, when expressed as a Y2H prey construct. Haploid yeast were initially mated on YPAD media for 24 h prior to replication onto SD-Leu/-Trp diploid selection media and grown for 48 h. Protein-protein interaction was detected following the replication of diploid yeast onto the above media also lacking adenine or histidine (supplemented with 2.5 mM 3-AT). Activation of reporter genes *ADE2* and *HIS3* and thus growth, indicative of protein-protein interaction, was scored over a period of 14 days. Only interactions reproducibly observed in two independent assays were scored as positive interactions.

#### 4.3.3 Generation of KEAP1^−/−^, NRF2^−/−^, and NRF2^T80K^ HEK293T cell-lines

All cell-lines were generated using CRISPR/Cas9 technology. For NRF2^−/−^ and KEAP1^−/−^, HEK293T cells were seeded in 12-well plates (1 × 10^5^ cells/well) 24 h prior to transfection. Cells were transfected with 1 μg LentiCRISPR guideRNA plasmids (NRF2, 5′-TTACAACTAGATGAAGAGAC-3’; KEAP1, 5′-GCCAGATCCCAGGCCTAGCG-3′) using Lipofectamine-2000 (Life Technologies). After 24 h incubation, the medium was changed to include 2 μg/mL puromycin (Sigma) and the cells were grown for an additional 48 h before limiting dilution and selection of positive clonal populations. For NRF2^T80K^, HEK293T cells were seeded in a 96-well plate (1 × 10^3^ cells/well) 24 h prior to transfection. Cells were co-transfected with 10 pmol Cas9 RNP (Synthego) and 100 pmol HDR template supplied as an ssODN (Sigma; 5′-AAGACAAGAACAACTCCAAAAGGAGCAAGAGAAAGCCTTTTTCGCTCAGTT ACAACTAGACGAGGAAAAAGGAGAATTCCTCCCAATTCAGCCAGCCCAGCACATCCAGTCAGAAACCAGTGGATCTGCCAACTACTCC-3′) for 48 h prior to limiting dilution and selection of positive clonal populations.

#### 4.3.4 Co-immunoprecipitation

HEK293T cells were co-transfected with 1 μg each of EGFP-tagged NRF2 and mCherry-tagged binary partner vectors for 24 h. Cells were lysed in NP-40 lysis buffer (0.5% NP-40 substitute, 25 mM Tris-HCl pH 7.5, 150 mM NaCl, 2 mM MgCl_2_, 1 mM EGTA, protease and phosphatase inhibitor cocktail), following which clarified protein lysates were incubated with GFP-Trap agarose beads (ChromoTek) for 2 h at 4 °C, with rotation. Beads were collected by centrifugation and washed thrice in NP-40 lysis buffer, then once in IP wash buffer (10 mM Tris pH 7.5, 2 mM MgCl_2_). Proteins were eluted directly with NuPAGE LDS Sample Buffer (Invitrogen) for 10 min at 80 °C before being resolved by SDS-PAGE and Western blotting.

#### 4.3.5 FCCS quantification

FCCS data collection was performed as previously described [41], using a 63x/1.4 Plan-Apochromat objective on a Zeiss LSM 780. Briefly, HEK293T cells were seeded overnight in four-compartment glass bottom dishes (2.5 × 10^4^ cells/compartment) and transfected with 25 ng of EGFP-tagged NRF2 and mCherry-tagged binary partner for 24 h. EGFP fluorescence was excited using a 488 nm Argon laser and emission collected between 500 and 530 nm, while mCherry fluorescence was excited using a 561 nm DPSS diode and emission collected between 590 and 640 nm. Data from individual cells were collected via 5 × 2 s measurements either in the cytoplasm or nucleus dependent upon partner localisation using 0.15–0.3% laser power to minimise bleaching and a suitable count rate of approximately 1 kHz count rate per molecule (CPM). The autocorrelation G_auto_(τ) and cross-correlation G_cross_(τ) functions were calculated using the ZEN 2012 software. The calculated autocorrelation and cross-correlation values were fitted to a two-component diffusion model with or without triplet state correction respectively using the Levenberg-Marquardt algorithm in MATLAB optimisation toolbox. The *in vivo* K_d_ values were obtained from nonlinear fitting by plotting the fraction of bound EGFP-tagged and mCherry-tagged proteins as a function of free mCherry-tagged or EGFP-tagged proteins respectively, and averaging the fitted values.

#### 4.3.6 DULIP quantification

DULIP was modified from previously described [45]. Briefly, HEK293T cells were transfected for 24 h with 25 ng each of *Renilla* luciferase (RL)-tagged NRF2 and firefly luciferase (FL)-tagged binary partner vectors. Following transfection for 24 h, cells were lysed in DULIP lysis buffer (50 mM Tris-HCl pH 7.4, 150 mM NaCl, 10% glycerol, 1% NP-40, 0.25% sodium deoxycholate, 1 mM EDTA, 1 mM DTT, protease and phosphatase inhibitor cocktail) and transferred to 96-well plates pre-coated with rabbit IgG (Sigma) in carbonate-bicarbonate coating buffer (pH 9.6) and blocked with 1% bovine serum albumin for 3 h at 4 °C. Firefly and *Renilla* luminescence were measured sequentially using a GloMax Multi-Detection System (Promega).

#### 4.3.7 ARE-luciferase activity screens

HEK293T cells were co-transfected for 24 h using 100 ng of 8XARE-FL, 5 ng of *Renilla* luciferase (pRL-SV40), as well as 100 ng of an expression vector containing the binary partner in a 96-well plate. Cells were lysed in Passive Lysis Buffer (Promega) and transferred to a 96-well plate before firefly and *Renilla* luminescence were measured sequentially on a GloMax Multi-Detection System. Log2-normalised luciferase fold change was fitted to a linear mixed model with ‘condition’ and ‘cell-line’ as fixed effects and ‘plate’ as a random effect using the ‘nlme’ [53] and ‘lmerTest’ [54] R packages. p-values were calculated by Satterthwaite's method for unequal variance and corrected for multiple testing using Bonferroni's method.

#### 4.3.8 Network construction

Protein interaction data was extracted and merged from BioGRID [33,34], IntAct [48], HuRI [50], HPRD [49], and innateDB [51]. The predicted binary NRF2 network was generated as previously described [30]. To increase network density we also included: (i) additional mammalian interologs from BioGRID [33,34], (ii) interologs involving the *C. elegans* NRF2 ortholog SKN-1 in Wormbase [35], WormNet [36] and the CCSB Worm ORFeome [37], (iii) interologs from the *Drosophila melanogaster* ortholog CncC in Flybase [38] and BioGRID databases, as well as (iv) predictions from the Reactome Functional Interactome (ReactomeFI) [39]. Gene-disease association terms were extracted from DisGeNET [4] using a cut-off ≥0.2. Networks were generated and visualised using Cytoscape 3.7.1.

## Data and code availability

MATLAB custom analysis code is available from the lead contact upon reasonable request. NRF2 protein-protein interaction data have been submitted to the IMEx (http://www.imexconsortium.org) consortium through IntAct [48] (IMEx ID: IM-27648).

## Author contributions

JP, AHP, JB contributed experimental data. CMS, AHP, JP, JB, DM, NH contributed to experimental design. US, EW, JDW contributed to clone generation and development of assay reagents. All authors contributed to manuscript preparation.

## Declaration of competing interest

The authors declare no competing interest.

## Appendix A Supplementary data

Multimedia component 1 Multimedia component 1

Supplementary Table S1 Supplementary Table S1 Predicted binary NRF2 interaction partners. Related to Fig. 1. Supplementary Table S2 Plasmid constructs. Related to Materials and Methods. Supplementary Table S3 Disease-association terms for NRF2 interaction partners. Related to Fig. 5. Novel partners are highlighted in bold. Supplementary Table S1

## Footnotes

Appendix A Supplementary data to this article can be found online at https://doi.org/10.1016/j.redox.2020.101686.
