# Supplementary material for: A functionally defined high-density NRF2 interactome reveals new conditional regulators of ARE transactivation
Source: Redox Biol. 2020 Aug 20;37:101686. doi: 10.1016/j.redox.2020.101686 (PMC7490560; doi:10.1016/j.redox.2020.101686)
Supplement: Multimedia component 1 [file mmc1.docx]

**Figure S1. Related to Figure 1.**

Representative immunoblots used to generate Figure 1C. EGFP-tagged NRF2 was transiently co-expressed with mCherry-tagged binary partners in HEK293T cells and co-immunoprecipitated with GFP-Trap agarose beads. Blots are representative of 3 independent experiments. Input represents 20 μg of cell lysate.

**Figure S2. Related to Figure 2.**

Representative images of cells co-expressing EGFP-tagged NRF2 and mCherry-tagged binary

partners. The locations where FCS measurement were recorded are marked with cross-hairs, corresponding to the areas of partner co-localisation.

**Figure S3. Related to Figure 2.**

FCCS *in vivo* K_d_ plots used to generate Figure 2A. Fraction of bound mCherry-tagged binary partners or EGFP-tagged NRF2 plotted against free EGFP-NRF2 or free mCherry-tagged binary partner, fitted to a one-site binding curve. Each point represents a single cell measurement.

**Figure S4. Related to Figure 2.**

(A) FCCS K_d_ values are plotted against DULIP K_d_ values. Linear fit equation allows for calibration of DULIP K_d_ values (**Supplementary Methods**), *** indicates *p* ≤ 0.001.

(B) *Renilla*-tagged (RL)-NRF2 and 35 firefly-tagged (FL)-partners were co-expressed as pairs in KEAP1^-/-^ cells and interactions were quantified by DULIP. Binding strength is expressed as scaled DULIP K_d_ values ± SEM, n ≥ 9 measurements from ≥3 independent experiments. (C) Scaled DULIP K_d_ values measured in HEK293T cells plotted against scaled DULIP K_d_ values measured in KEAP1^-/-^ cells. Broken lines represent a 1.25-fold margin between the two K_d_ values. Interaction partners with K_d_ values differing >1.25-fold in the two cell-lines are highlighted.

**Figure S5A&B. Related to Figure 3.**

(A) The effect of selective NRF2 Neh domain deletion (dNeh) on the ability of MAFG to bind to NRF2 was assessed using the DULIP assay system; cNIR, corrected normalised interaction ratio; N = 3 independent experiments in triplicates. (B) Results from a preliminary screen to investigate the effects of phospho-modulation at 6 predicted phosphorylation sites within NRF2. Relative fold change of ARE-luciferase activity following 24 h transfection of EGFP-tagged NRF2 WT or mutated forms of NRF2 are displayed; N = 2 independent experiments in triplicates.

**Figure S6. Related to Figure 5.**

(A) Disease-gene association network for NRF2 using a cut-off ≥0.2 based on the DisGeNET tool. Transcription factors are bordered in red, bZIP TFs in pink, kinases in light green, and ubiquitin-related proteins in dark green. Previously known partners are shaded in light blue and novel partners from this study in dark blue. Diseases nodes are shaded green if representing a neoplastic process, yellow if representing a mental or behavioural process, red if representing a disease or a syndrome, and pink for other abnormalities. Edges represent gene-disease interactions.

(B) Disease-centric sub-networks. PPI edges are in dark grey and gene-disease edges in light grey.

**Supplementary Methods. Calculation of K_d_ values. Related to Figure 2.**

FCS/FCCS Correlation Curves

The calculated auto- and cross-correlation curves ($G\left( \tau\right)$) were fitted to a two-component diffusion with or without triplet state correction respectively given by,

$$G\left( \tau\right)=G\left( 0 \right)\cdot\frac{1}{\left( 1-T \right)}\cdot\left[ 1-T\cdot\left( 1- e^{\frac{-\tau}{\tau_{T}}} \right) \right]\cdot\sum_{i=1}^{2} \left[ a_{i}\cdot\left( 1+\frac{\tau}{\tau_{D_{i}}} \right)^{-1}\cdot\left( 1+\frac{\tau}{\tau_{D_{i}}{\cdot S}^{2}} \right)^{-\frac{1}{2}} \right]$$

where $\tau$ represents the autocorrelation lag time, $G\left( 0 \right)$ corresponds to the amplitude of the correlation curve, $T$ represents the fraction of molecules in the triplet state, $\tau_{T}$ represents the t triplet lifetime, $a_{i}$ represents the faction of each diffusion component (e.g. fast and slow), $\tau_{D_{i}}$represents the diffusion time for each component, and $S={Z_{0}}/{\omega_{0}}$ is the structural parameter determined by the ratio of the axial ($Z_{0}$) to lateral ($\omega_{0}$) dimensions of the detection volume previously calibrated to give $S=4$.

FCCS K_d_ Equations

For the green channel, i.e. EGFP-NRF2 measurements, the relative cross-correlation ($RCC_{green}$) can be used to calculate dissociation constant ($K_{d}$) by,

$$\frac{\left[ Complex \right]}{\left[ Nrf2_{total} \right]}=\frac{\left[ Partner_{total} \right]-\left[ Complex \right]}{K_{d}+\left[ Partner_{total} \right]-\left[ Complex \right]}$$

and for the red channel, i.e. mCherry labelled partners, by

$$\frac{\left[ Complex \right]}{\left[ Partner_{total} \right]}=\frac{\left[ Nrf2_{total} \right]-\left[ Complex \right]}{K_{d}+\left[ Nrf2_{total} \right]-\left[ Complex \right]}$$

with $\left[ \right]$ denoting molar concentration of total EGFP and mCherry species given by,

$$\left[ X \right]=\frac{1}{G\left( 0 \right)}\cdot\frac{1}{N_{A}}\cdot\frac{1}{V_{eff}}$$

and for the red-green complexed proteins by,

$$\left[ Complex \right]=\frac{G_{cross}\left( 0 \right)}{G_{green}\left( 0 \right)\cdot G_{red}\left( 0 \right)}\frac{1}{N_{A}}\frac{1}{V_{eff}}$$

where $N_{A}$ represents Avogadro’s constant and $V_{eff}$represents the effective confocal volume previously measured to be 0.57 fL.

DULIP Values

The corrected normalised interaction ratio ($cNIR$) was calculated as follows,

$$cNIR=\frac{\frac{FL}{RL}-\frac{FL}{RL_{neg}}}{\frac{FL}{RL_{pos}}}$$

where $RL$ denotes *Renilla* luminescence tagged Nrf2, $FL$ firefly luciferase tagged partner, and $neg$and $pos$correspond to negative mCherry control and a positive *Renilla*-firefly fusion control respectively.

DULIP K_d_ Equations

$K_{d}$can be defined as,

$$K_{d}=\frac{\left( \left[ Nrf2_{total} \right]-\left[ Complex \right] \right)\cdot(\left[ Partner_{total} \right]-[Complex])}{[Complex]}$$

Given $f_{p}=\left[ Complex \right]/[Partner_{total}]$ i.e. the fraction of bound partner, $K_{d}$can be expressed as,

$$K_{d}=\left( \frac{1-f_{p}}{f_{p}} \right)\cdot(\left[ Nrf2_{total} \right]-[Complex])$$

Using Nrf2 as bait (RL tagged), $f_{p}$can be equated to,

$$f_{P}=\frac{FL-FL_{neg}}{FL_{pos}-FL_{neg}}$$

and free NRF2 can be calculated by,

$$\left[ Nrf2_{total} \right]-\left[ Complex \right]=RL_{test}-\frac{FL}{\frac{FL_{pos}}{RL_{pos}}}$$

allowing for the calculation of DULIP K_d_ in arbitrary units by,

$$DULIP K_{d}=\left( \frac{1-f_{p}}{f_{p}} \right)\cdot\left( RL-\frac{FL}{\frac{FL_{pos}}{RL_{pos}}} \right)$$

DULIP K_d_ values were then calibrated to units of nM by comparison to FCCS data according to the equation,

$$\log\left| FCCS K_{d} \right| =\beta\log\left| DULIP K_{d} \right|+\alpha$$

where the constants $\alpha$ and $\beta$ were determined by comparing FCCS and DULIP K_d_ values across interacting partners.
